# The association between daytime napping and risk of type 2 diabetes is modulated by inflammation and adiposity: Evidence from 435 342 UK‐Biobank participants

**DOI:** 10.1111/1753-0407.13387

**Published:** 2023-04-13

**Authors:** Rui Zhou, Hao‐Wen Chen, Yi‐Ning Huang, Qi Zhong, Fu‐Rong Li, Rui‐Dian Huang, Hua‐Min Liu, Jia‐Zhen Zheng, Jia‐Wen Xu, Xian‐Bo Wu

**Affiliations:** ^1^ Department of Epidemiology, School of Public Health (Guangdong Provincial Key Laboratory of Tropical Disease Research) Southern Medical University Guangzhou China; ^2^ School of Medicine Southern University of Science and Technology Shenzhen China; ^3^ Public Health Division Hospital of Zhongluotan Town Baiyun District Guangzhou China; ^4^ Department of Anesthesiology, Nanfang Hospital Southern Medical University Guangzhou China; ^5^ Bioscience and Biomedical Engineering Thrust, Systems Hub The Hong Kong University of Science and Technology (Guangzhou) Guangzhou China; ^6^ Bioscience and Biomedical Engineering Thrust, Systems Hub The Hong Kong University of Science and Technology Hong Kong China; ^7^ School of Public Health Southern Medical University Guangzhou China

**Keywords:** adiposity, cohort, daytime napping, inflammation, type 2 diabetes, 脂肪, 队列研究, 午睡, 炎症, 2型糖尿病

## Abstract

**Background:**

Existing evidence concerning the relationship between daytime napping and type 2 diabetes (T2D) is inconsistent, and whether the effects of napping differ by body fat percentage (BFP) and C‐reactive protein (CRP) is unclear. We aimed to investigate the association between daytime napping frequency and T2D risk and whether such an association was modified by BFP and CRP.

**Methods:**

We included 435 342 participants free of diabetes from the UK Biobank. Participants were categorized as nonnappers, occasional nappers, and frequent nappers based on napping frequency, and BFP/CRP was divided into quartiles. Cox proportional hazards models were used.

**Results:**

During a median follow‐up of 9.2 years, 17 592 T2D cases occurred. Higher frequency of daytime napping was significantly associated with an increased risk of T2D. Compared with nonnappers, the adjusted hazard ratios (HRs) for occasional nappers and habitual nappers were 1.28 (95% confidence interval [CI]: 1.24–1.32) and 1.49 (95% CI: 1.41–1.57), respectively. There was a significant additive and multiplicative interaction (relative excess risk due to interaction [RERI] = 0.490, 95% CI 0.307–0.673; *p* for multiplicative interaction <.001) between napping and BFP, whereby a higher hazard of T2D associated with more frequent napping was greatest among participants in the highest BFP quartile (HR = 4.45, 95% CI: 3.92–5.06). The results for CRP were similar (RERI = 0.266, 95% CI: 0.094–0.439; *p* for multiplicative interaction <.001).

**Conclusions:**

Higher daytime napping frequency is associated with an increased T2D risk, and such relationships are modified by BFP and CRP. These findings underscore the importance of adiposity and inflammation control to mitigate diabetes risk.

## Graphical abstract

**Highlights**

- Using a large‐scale prospective population‐based cohort study, we found that a higher frequency of daytime napping is associated with an increased risk of incident type 2 diabetes (T2D), and such relations are modified by body fat percentage (BFP) and C‐reactive protein (CRP) levels. The association between daytime napping and T2D risk is strongest among those with the lowest BFP and CRP levels, which provides implications for the development of T2D prevention strategies targeting the reduction in BFP and CRP levels among adults with napping habits.

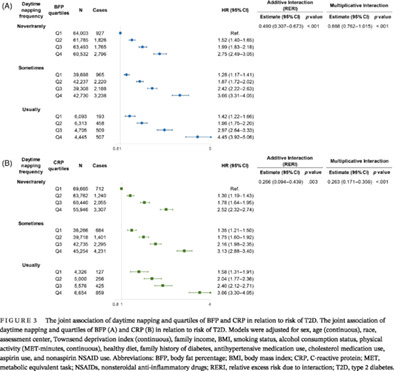

## 摘要

**【摘要】 背景** 关于午睡与2型糖尿病之间的关系，现有的证据不一致，并且午睡的效应是否会受到体脂百分比(BFP)和C‐反应蛋白(CRP)的影响尚不清楚。本研究旨在调查午睡频率与2型糖尿病风险之间的关联性，以及这种关联是否受到BFP和CRP的调节。**方法** 本研究纳入了来自英国生物银行(UK‐Biobank)的435 342名无糖尿病的参与者。根据午睡频率，参与者被分为不午睡者、偶尔午睡者和经常午睡者，并将BFP/CRP分为四分位数。采用*Cox*比例风险模型进行分析。**结果** 在中位随访9.2年期间，共发生2型糖尿病17 592例。白天午睡频率越高，2型糖尿病患病风险也越高。与不午睡者相比，偶尔午睡者和经常午睡者的校正后风险比(*HR*)分别为1.28(95% *CI* 1.24~1.32)和1.49(95% *CI* 1.41~1.57)。午睡与BFP之间存在显著的加性和乘性交互作用[交互作用的相对过度风险(*RERI*)=0.490，95% *CI* 0.307~0.673, 乘性交互作用的*P*<0.001]，即与频繁午睡相关的2型糖尿病风险增加在最高BFP四分位数的参与者中最为显著(*HR*=4.45，95% *CI* 3.92~5.06)。CRP的结果也类似(*RERI*=0.266，95% *CI* 0.094~0.439, 乘性交互作用的*P*<0.001)。**结论** 午睡频率越高，2型糖尿病风险也越高，并且这种关系受到BFP和CRP的调节。这些发现强调了控制脂肪堆积和炎症对降低糖尿病风险的重要性。

## 1 INTRODUCTION

Daytime napping, defined as a short sleep typically taken in the early afternoon, is a common habit and lifestyle practice in many regions around the world.^1^ Although some acute benefits have been attributed to napping, such as compensating for sleep deprivation^2^, ^3^ and improving work efficiency and mental health in daily functioning, the long‐term effects of habitual napping on chronic disease risk remain controversial. Notably, the relationship between daytime napping and type 2 diabetes (T2D) has been investigated in numerous recent studies. However, the results of different studies are conflicting. Several studies have shown that daytime napping was associated with an increased risk of T2D, especially in the Mediterranean region, Asia, and Central America.^4^, ^5^, ^6^ Conversely, in other studies, no significant relationship between the two was observed.^7^, ^8^ Another Chinese study among middle‐aged and older adults even demonstrated that short daytime napping (<1 h/day) could reduce T2D risk.^9^ The inconsistent associations might be partly due to the modification effects of varying metabolic risk factors related to daytime napping across the studies.

Emerging evidence has linked inflammation status and adiposity with daytime napping. Subjects with longer napping durations were found to have an increased level of interleukin‐6,^10^ and those who reported having a habit of napping had increased C‐reactive protein (CRP) levels.^11^ This phenomenon is possibly because awakening from long naps triggers a rise in blood pressure or heart rate, resulting in increased endothelial shear stress and immune response.^12^ In addition, previous studies suggest that long daytime napping durations were positively associated with adiposity measures, including waist circumference and body mass index (BMI),^13^, ^14^ which could be explained by the fact that napping may promote hunger and appetite, and the cortisol levels may increase after naps, which, in turn, leads to fat accumulation.^15^ Significantly, both inflammation and adiposity have also been related to an increased risk of T2D.^16^, ^17^, ^18^ Therefore, we hypothesized that higher frequency daytime napping might be associated with an increased risk of T2D and that inflammation and adiposity might modify the association. In this study, we prospectively investigated the association of daytime napping frequency with the risk of T2D among 435 342 adults from the UK Biobank, a large‐scale, prospective, population‐based cohort. We also examined the potential effect modification of CRP level, the most commonly studied systemic marker of chronic inflammation,^19^ and body fat percentage (BFP), which is adopted as a better predictor to evaluate the amount of body fat content,^20^, ^21^ on the associations.

## 2 METHODS

### 2.1 Study population

The UK Biobank is a large‐scale and prospective cohort study with over 500 000 participants aged 40–69 years recruited from 2006 to 2010 from across the United Kingdom (www.ukbiobank.ac.uk).^22^ Participants attended one of 22 assessment centers across England, Scotland, and Wales, where they completed touchscreen and face‐to‐face questionnaires, underwent physical measurements, provided biological samples and were longitudinally followed up for a wide range of health‐related outcomes. All participants gave written consent, and ethical approval was obtained from the Northwest Multicenter Research Ethics Committee (London, UK)

**FIGURE 1:**
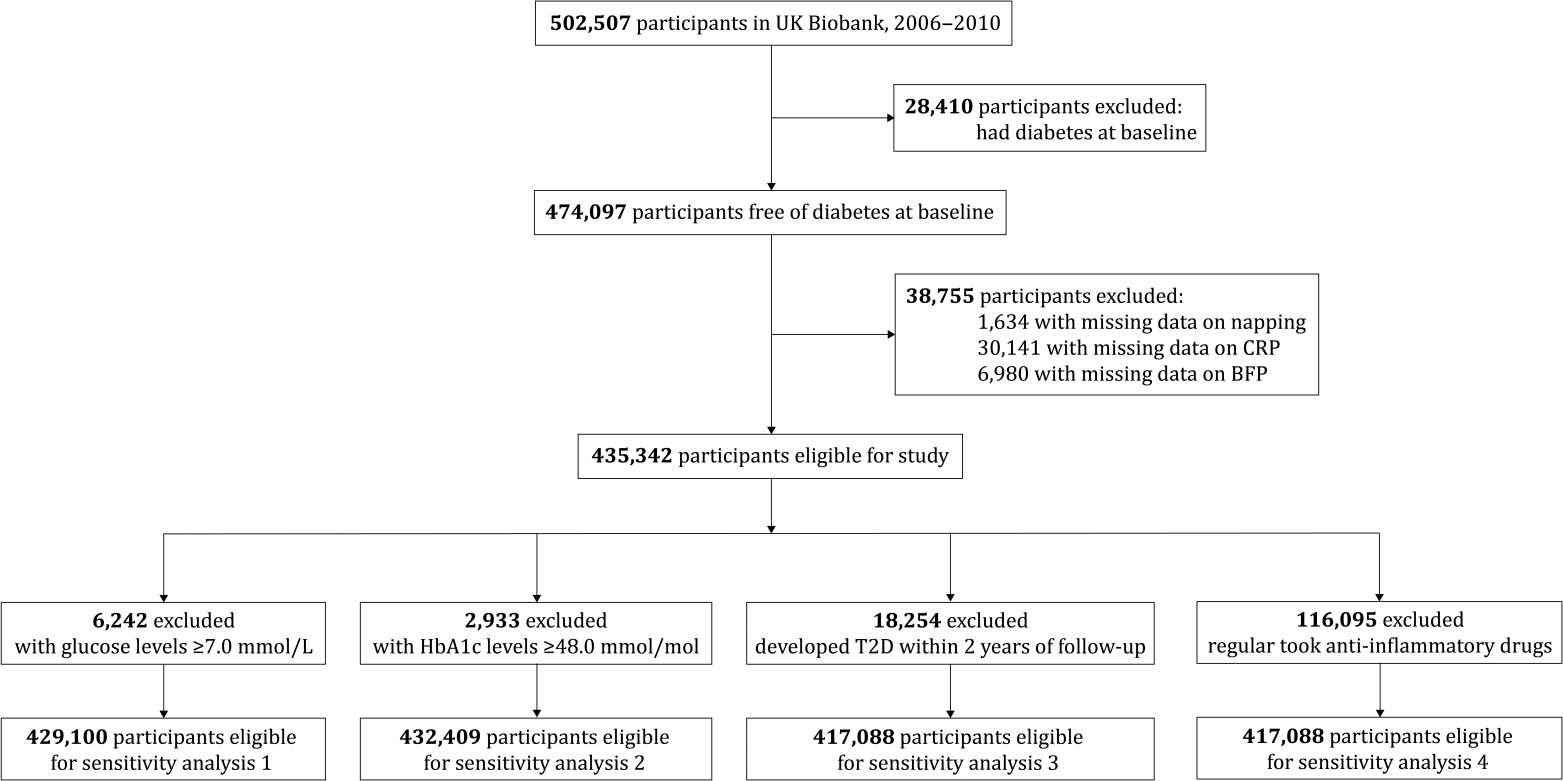
Study flow chart. BMI, body mass index; CRP, C‐reactive protein; HbA1c, glycosylated hemoglobin; T2D, type 2 diabetes.

In the current study, we excluded individuals with diabetes at baseline (*n* = 28 410) and those with missing data on daytime napping (*n* = 1634), CRP (*n* = 30 141), or BFP (*n* = 6980) at baseline, leaving a total of 435 342 participants in the main analysis (Figure 1).

### 2.2 Exposure assessment

At the baseline assessment, all study participants reported their daytime napping frequency through a touch‐screen questionnaire by answering the following question: “Do you have a nap during the day?” with the responses being (a) never or rarely (< once per week), (b) sometimes (1–3 times per week), or (c) usually (≥4 times per week), where every category increase reflected more frequent napping. Prefer not to answer responses were set to missing.

Blood samples were collected from the participants at the initial assessment center visit (2006–2010). CRP (mg/L) was measured by immunoturbidimetric high‐sensitivity analysis on a Beckman Coulter AU5800, glucose (mmol/L) was measured by hexokinase analysis on a Beckman Coulter AU5800, and glycosylated hemoglobin (HbA1c; mmol/mol) was measured by high‐performance liquid chromatography analysis on a Bio‐Rad VARIANT II Turbo. Details on quality control and sample preparation have been described previously.^23^ CRP was log‐transformed in all analyses due to its skewed distribution.

Body composition measures of all participants were assessed by trained personnel using a standard protocol at enrollment. BFP was estimated using a Tanita BC418MA segmental body composition analyzer (Tanita, Tokyo, Japan) according to the manufacturer's instructions. This device estimates body composition by conducting bioimpedance analysis. Both log (CRP) and BFP levels were treated as continuous variables. In addition, participants were grouped based on quartiles of log (CRP) and BFP to evaluate the effects of these variables on the association between daytime napping and incident T2DM.

### 2.3 Ascertainment of outcomes

The UK Biobank algorithms^24^ used to define incident and prevalent outcomes have been reported (Supplementary Table S1 in Appendix S1). In brief, prevalent diabetes was ascertained according to medical history and medication use self‐reported and queried by trained health professionals at baseline; incident T2D was ascertained using admissions and diagnoses data of hospital inpatient records obtained through linkage to Hospital Episode Statistics for England, Scottish Morbidity Record data for Scotland, and the Patient Episode Database for Wales, with the *International Classification of Diseases, Tenth Revision* code E11.

### 2.4 Statistical analysis

Baseline characteristics of the participants are described as the means (SDs) or *n* (percentage) according to the frequency of daytime napping (nerve/rarely, sometimes, or usually). The relationship between daytime napping and CRP levels, BFP levels, glucose levels, and HbA1c levels was evaluated using a general linear model. The associations of CRP, BFP, and daytime napping with incident T2D were investigated using Cox proportional hazard models. The proportional hazards assumption was tested using Schoenfeld residuals. Follow‐up time was calculated from the baseline date to diagnosis of T2D, death, loss to follow‐up, or censoring date (30 June 2020, which was the end of follow‐up for the current data release), whichever came first.

Two approaches were used. First, separate associations of daytime napping and quartiles of CRP and BFP with incident T2D were investigated. CRP and BFP were also treated as continuous variables, and hazard ratios (HRs) per one SD increase in inflammation and adiposity were estimated. Second, joint associations between daytime napping, CRP, and incident T2D and between daytime napping, BFP, and incident T2D were evaluated. Daytime napping frequencies and quartiles for CRP and BFP were derived, and HRs were calculated, with the referent category comprising individuals who were in both the lowest frequency group of daytime napping (never/rarely) and the highest quartile of CRP or BFP. To investigate whether adiposity and/or inflammation moderated the association between daytime napping and the risk of incident T2D, we assessed the relative excess risk due to interaction (RERI) as an index of additive interaction. In our study, RERIs were defined for dichotomized exposures^25^, ^26^ and were calculated by comparing frequent nappers to nonnappers using the formula $\left({\mathrm{HR}}_{\text{Frequent nappers},\text{highest}\;\mathrm{BFP}/\mathrm{CRP}\;\text{quartile}}-{\mathrm{HR}}_{\text{Frequent nappers},\text{lowest}\;\mathrm{BFP}/\mathrm{CRP}\;\text{quartile}}-{\mathrm{HR}}_{\text{Nonnappers},\text{highest}\;\mathrm{BFP}/\mathrm{CRP}\;\text{quartile}}\right)+1$. The confidence intervals (CIs) for RERI were estimated using the standard delta method.^27^ Multiplicative interaction was also tested by including a product term in the models.

Several potential confounders were adjusted in the models described: age (continuous), sex (male/female), race (white and others), UK Biobank assessment center, Townsend deprivation index (continuous), average total annual household income (<£18 000, £18 000–30 999, £31 000–51 999, £52 000–100 000, ≥£100 000, and “do not know” or missing), BMI (<18.5, 18.5–24.9 or 25–29.9, ≥30 kg/m^2^), smoking status (current, former, or never), alcohol consumption (current, former, or never), physical activity (metabolic equivalent task [MET]‐minutes per week, continuous), healthy diet (yes/no), family history of diabetes (yes/no), antihypertensive medication use (yes/no), cholesterol medication use (yes/no), aspirin use (yes/no), and nonaspirin nonsteroidal anti‐inflammatory drug (NSAID) use (yes/no). A healthy diet was defined as an adequate intake of at least four of seven commonly eaten food groups recommended as dietary priorities for cardiometabolic health^28^: fruit intake ≥3 servings/day; vegetable intake ≥3 servings/day; fish intake ≥2 servings/week; processed meat intake ≤1 servings/week; unprocessed red meat intake ≤1.5 servings/week; whole grain intake ≥3 servings/day; and refined grain intake ≤1.5 servings/day. Missing data were imputed with mean values for continuous variables or coded as a missing indicator category for categorical variables.

We performed a stratified analysis to evaluate potential modification effects according to the following factors: sex (male or female), race (White, Asian, Black, or mixed/other), age (<55 or ≥ 55 years), BMI (<30 or ≥ 30 kg/m^2^), physical activity (<600 MET min/week or ≥ 600 MET min/week), healthy diet (yes or no), sleep duration (short [<6 h/night], normal [6–9 h/night], long [>9 h/night]), smoking status (never, past, current), alcohol consumption status (never, past, current), Townsend deprivation index (<median or ≥ median), family history of diabetes (yes or no), aspirin use (yes or no), and nonaspirin NSAID use (yes or no). Sleep duration was based on the following standardized question: “Approximately how many hours do you sleep every 24 h?”, for which responses were given in hours (https://bbams.ndph.ox.ac.uk). Sleep duration information was considered to be missing if participants failed to answer or had a sleep duration <4 or > 18 h to reduce the possibility of an implausible sleep duration. Based on the classification proposed by the National Sleep Foundation,^29^, ^30^ sleep duration was categorized as short (<6 h/night), normal (6–9 h/night), and long (>9 h/night). The interaction test between daytime napping (usually versus never/rarely) and each category was performed by adding interaction terms to the Cox models. Several sensitivity analyses were also performed to confirm the robustness of our results^1^: excluding participants with glucose levels ≥7.0 mmol/L (*n* = 6242) or HbA1c levels ≥48 mmol/mol (6.5%, *n* = 2933) to address the issues of potential undiagnosed diabetes at baseline^2^; excluding participants with T2D incidence occurring in the first 2 years to minimize the influence of reverse causation (*n* = 18 254)^3^; excluding participants who regularly took an anti‐inflammatory drug (aspirin or nonaspirin NSAID) to minimize the influence of other regular inflammatory drug use (*n* = 116 095); and^4^ adjusting for glucose levels, HbA1c levels, CRP levels, or sleep duration (hours) in the multivariable models.

All analyses were conducted using Stata (version 15; StataCorp, College Station, Texas). All statistical tests were two sided, and *p* < .05 was considered statistically significant.

## 3 RESULTS

### 3.1 Baseline characteristics of participants by daytime napping frequency

Table 1 shows the baseline characteristics of the study participants stratified by the frequency of daytime napping. Of the 435 342 participants (mean [SD] age, 56.3 (8.1) years), 240 665 (55.3%) were women. Overall, 249 813 (57.4%) participants reported never or rarely napping during the day (nonnappers), 163 973 (37.7%) reported napping sometimes (occasional nappers), and 21 556 (5.0%) reported napping often (habitual nappers) at baseline.

**TABLE 1:** Baseline characteristics of participants by frequency of daytime napping in the UK Biobank cohort.

Characteristics	Overall	Daytime napping		
		Never/rarely	Sometimes	Usually
*N*	435 342	249 813 (57.4)	163 973 (37.7)	21 556 (5.0)
CRP (mg/L)	2.5 (4.2)	2.3 (4.0)	2.8 (4.5)	3.1 (5.2)
BFP (%)	31.3 (8.5)	31.2 (8.5)	31.6 (8.6)	30.4 (8.5)
Age (years)	56.3 (8.1)	55.4 (8.1)	57.5 (7.9)	59.1 (7.6)
Female	240 665 (55.3)	148 967 (59.6)	84 078 (51.3)	7620 (35.4)
White ethnicity	387 579 (89.0)	223 244 (89.4)	145 336 (88.6)	18 999 (88.1)
Townsend deprivation index	−1.4 (3.0)	−1.5 (3.0)	−1.2 (3.1)	−0.9 (3.3)
Household income (£)				
< 18 000	81 685 (18.8)	39 402 (15.8)	36 184 (22.1)	6099 (28.3)
18 000–30 999	95 493 (21.9)	51 576 (20.7)	38 594 (23.5)	5323 (24.7)
31 000–51 999	99 595 (22.9)	60 062 (24.0)	35 244 (21.5)	4289 (19.9)
52 000–100 000	78 604 (18.1)	51 979 (20.8)	24 300 (14.8)	2325 (10.8)
≥ 100 000	21 025 (4.8)	14 677 (5.9)	5889 (3.6)	459 (2.1)
BMI (kg/m^2^)				
< 18.5	2112 (0.5)	1363 (0.6)	632 (0.4)	117 (0.5)
18.5–24.9	144 900 (33.3)	91 843 (36.8)	47 531 (29.0)	5526 (25.6)
25–29.9	188 701 (43.4)	106 401 (42.6)	72 746 (44.4)	9554 (44.3)
≥ 30	99 622 (22.9)	50 200 (20.1)	43 063 (26.3)	6359 (29.5)
Smoking status				
Never	239 899 (55.1)	144 047 (57.7)	85 819 (52.3)	10 033 (46.5)
Former	148 627 (34.1)	81 727 (32.7)	58 576 (35.7)	8324 (38.6)
Current	45 396 (10.4)	23 316 (9.3)	19 970 (11.6)	3110 (14.4)
Alcohol consumption status				
Never	17 705 (4.1)	9439 (3.8)	7149 (4.4)	1117 (5.2)
Former	14 521 (3.3)	7057 (2.8)	6189 (3.8)	1275 (5.9)
Current	402 667 (92.5)	233 092 (93.3)	150 445 (91.8)	19 130 (88.8)
MET (minutes/week)	12759.0 (12974.2)	12735.7 (12796.4)	12755.6 (13017.8)	13055.1 (14584.8)
Healthy diet	178 431 (41.0)	105 537 (42.3)	64 828 (39.5)	8066 (37.4)
Family history of diabetes	71 597 (16.5)	40 966 (16.4)	27 170 (16.6)	3461 (16.1)
Medication use				
Antihypertensive medications	85 716 (19.7)	41 475 (16.6)	37 907 (23.1)	6334 (29.4)
Cholesterol‐lowering medications	68 659 (15.8)	32 808 (13.1)	30 474 (18.6)	5377 (24.9)
Aspirin	54 516 (12.5)	25 955 (10.4)	24 127 (14.7)	4434 (20.6)
Nonaspirin NSAIDs	69 424 (16.0)	39 586 (15.9)	26 679 (16.3)	3159 (14.7)

*Note*: Data are presented as the mean (SD) for continuous variables and n (%) for categorical variables.

Abbreviations: BFP, body fat percentage; BMI, body mass index; CRP, C‐reactive protein; MET, metabolic equivalent task; NSAIDs, nonsteroidal anti‐inflammatory drugs.

After adjustment for a set of covariates, daytime napping frequencies showed a significant positive association with glucose, HbA1c, BFP and CRP levels at baseline (Supplementary Table S2 in Appendix S1).

### 3.2 Association between daytime napping and risk of incident T2D

During a median of 9.2 years of follow‐up (4 013 849 person‐years), 17 592 incident cases of T2D were identified. Kaplan–Meier curves of incident T2D demonstrated a significant, incremental increase in rates of incident T2D associated with increasing frequency of daytime napping (log‐rank *p* < .001, Supplementary Figure S1 in Appendix S1). Higher daytime napping frequency was significantly associated with an increased risk of incident T2D in the age‐, sex‐, and multivariate‐adjusted models (Table 2). In the age‐ and sex‐adjusted model, the HRs associated with daytime napping were 1.62 (95% CI: 1.57–1.67) and 2.15 (95% CI: 2.03–2.27) among occasional nappers and habitual nappers, respectively (*p* < .001). After further adjustment, the HRs were 1.28 (95% CI: 1.24–1.32) and 1.49 (95% CI: 1.41–1.57) among occasional nappers and habitual nappers, respectively.

**TABLE 2:** Adjusted HRs and 95% CI of daytime napping for T2D in the UK Biobank study.

	N	Events	Model 1	Model 2
Never/rarely	249 813	7314	Ref.	Ref.
Sometimes	163 973	8611	1.62 (1.57–1.67)	1.28 (1.24–1.32)
Usually	21 556	1667	2.15 (2.03–2.27)	1.49 (1.41–1.57)
*P* value^a^			<.001	

*Note*: Model 1 was adjusted for sex and age (continuous). Model 2 was additionally adjusted for race, assessment center, Townsend deprivation index (continuous), family income, BMI, smoking status, alcohol consumption status, physical activity (MET‐minutes, continuous), healthy diet, family history of diabetes, antihypertensive medication use, cholesterol medication use, aspirin use, and nonaspirin NSAID use.

^a^ *P* values were from chi‐square tests examining the difference in hazard ratio across groups.

Abbreviations: BMI, body mass index; CI, confidence interval; HR, hazard ratio; MET, metabolic equivalent task; NSAIDs, nonsteroidal anti‐inflammatory drugs; T2D, type 2 diabetes.

Similar associations of daytime napping with the risk of incident T2D were observed in several sensitivity analyses, including further removing participants with a high level of glucose (≥7.0 mmol/L), participants with a high level of HbA1c (≥48 mmol/mol [6.5%]), participants who developed T2D within 2 years of follow‐up or participants who took any anti‐inflammatory drugs or after adjusting for glucose levels, fasting time, HbA1c levels, or CRP levels (Supplementary Tables S3–S7 in Appendix S1).

**FIGURE 2:**
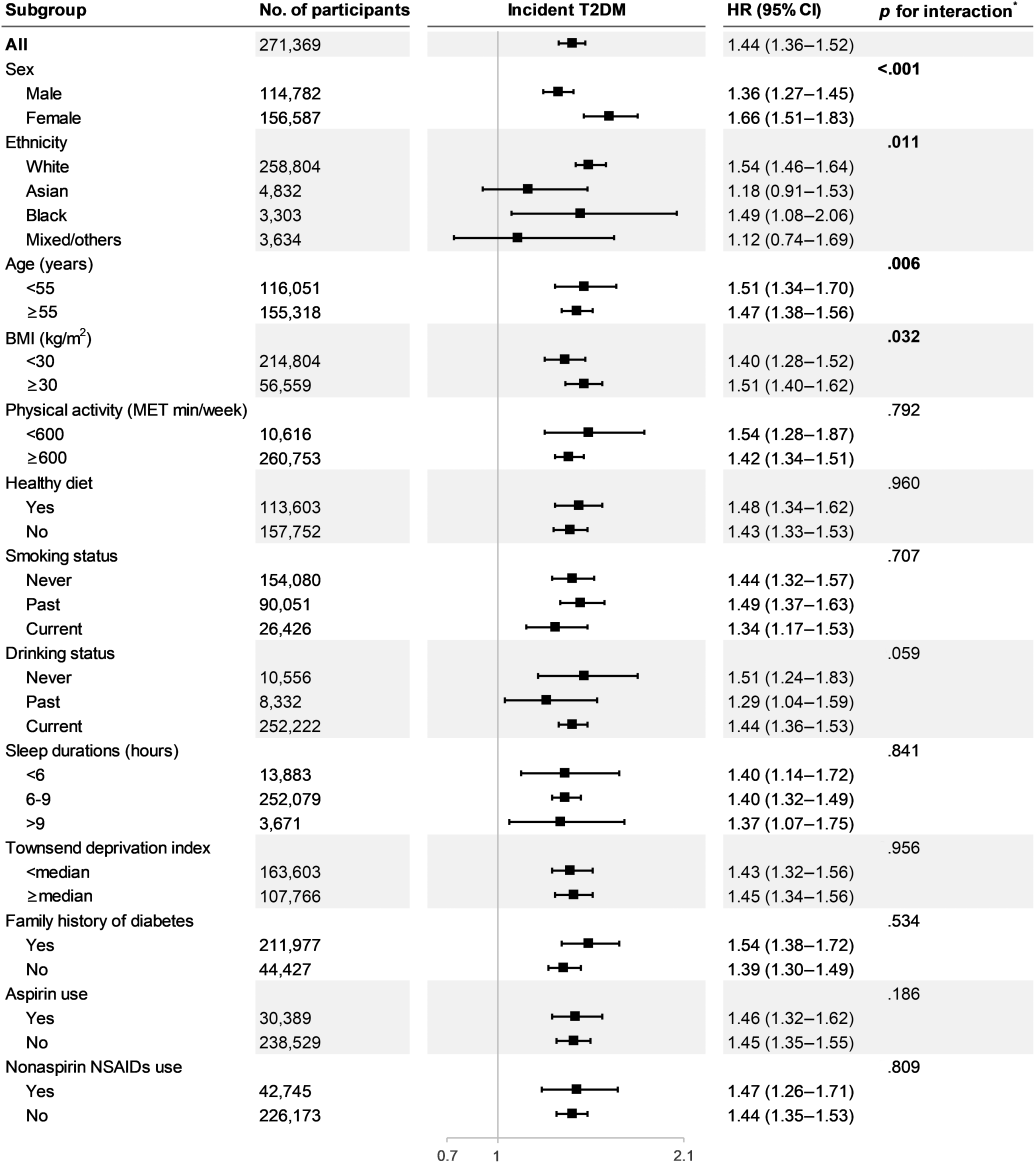
Adjusted HR (95% CI) for daytime napping (usually versus never/rarely) and risk of T2D stratified by potential risk factors. Models were adjusted for age, race, educational level, employment status, family income, marital status, social support construct scores, age at menopause, BMI, smoking status, alcohol consumption, physical activity, hypertension, diabetes mellitus, cardiovascular disease, depression, lipid‐lowering medication history, and hormone therapy treatment assignment. *The interaction test was performed by adding interaction terms to the Cox models. BMI, body mass index; CI, confidence interval; HR, hazard ratio; MET, metabolic equivalent task; NSAIDs, nonsteroidal anti‐inflammatory drugs; T2DM, type 2 diabetes mellitus.

We also conducted stratified analyses according to potential T2D risk factors to evaluate whether these factors modified the association between daytime napping and the risk of T2D (Figure 2). We found that the associations between daytime napping and the risk of incident T2D were stronger among men (*p* for interaction <.001), White and Black individuals (*p* for interaction = .011), younger participants (<55 years, *p* for interaction = .006) and obese participants (≥ 30 kg/m^2^, *p* for interaction = .032).

### 3.3 Interaction between daytime napping and CRP levels and BFP levels on the risk of T2D

We first evaluated the separate associations between inflammation, adiposity, and risk of incident T2D. Table 3 shows the HRs for the risk of T2D for quartiles of CRP and BFP levels. Higher levels of CRP and BFP were associated with an increased risk of incident T2D (all *p* for trend <.001), a one‐SD increase in CRP levels (1.1 mg/L) was associated with a 40% increase in the risk of incident T2D (95% CI: 37%–42%), and a one‐SD increase in BFP (8.5%) was associated with a 69% increase in the risk of incident T2D (95% CI: 64%–75%).

**TABLE 3:** Cox proportional hazard model of the association between inflammation, adiposity, and incidence of type 2 diabetes mellitus.

	*N*	Events	Model 1	Model 2
CRP (1‐SD increment)			1.76 (1.74–1.78)	1.40 (1.37–1.42)
Q1	110 257	1523	Ref.	Ref.
Q2	108 480	2897	1.80 (1.69–1.92)	1.30 (1.23–1.39)
Q3	108 751	4775	2.94 (2.78–3.12)	1.68 (1.58–1.78)
Q4	107 854	8397	5.44 (5.15–5.75)	2.43 (2.30–2.58)
*P* for trend^a^			<.001	<.001
Body fat percentage (1‐SD increment)			3.03 (2.97–3.10)	1.69 (1.64–1.75)
Q1	109 794	2085	Ref.	Ref.
Q2	110 335	4504	2.65 (2.51–2.79)	1.48 (1.40–1.57)
Q3	107 506	4462	5.47 (5.18–5.77)	1.97 (1.85–2.10)
Q4	107 707	6541	15.49 (14.56–16.48)	2.89 (2.66–3.15)
*P* for trend^a^			<.001	<.001

*Note*: Model 1 was adjusted for sex and age (continuous). Model 2 was additionally adjusted for race, assessment center, Townsend deprivation index (continuous), family income, BMI, smoking status, alcohol consumption status, physical activity (MET‐minutes, continuous), healthy diet, family history of diabetes, antihypertensive medication use, cholesterol medication use, aspirin use, and nonaspirin NSAID use.

Abbreviations: BMI, body mass index; CRP, C‐reactive protein; MET, metabolic equivalent task; NSAIDs, nonsteroidal anti‐inflammatory drugs.

^a^ The *p* value for trend was calculated using the median value in each quartile of CRP/BFP.

**FIGURE 3:**
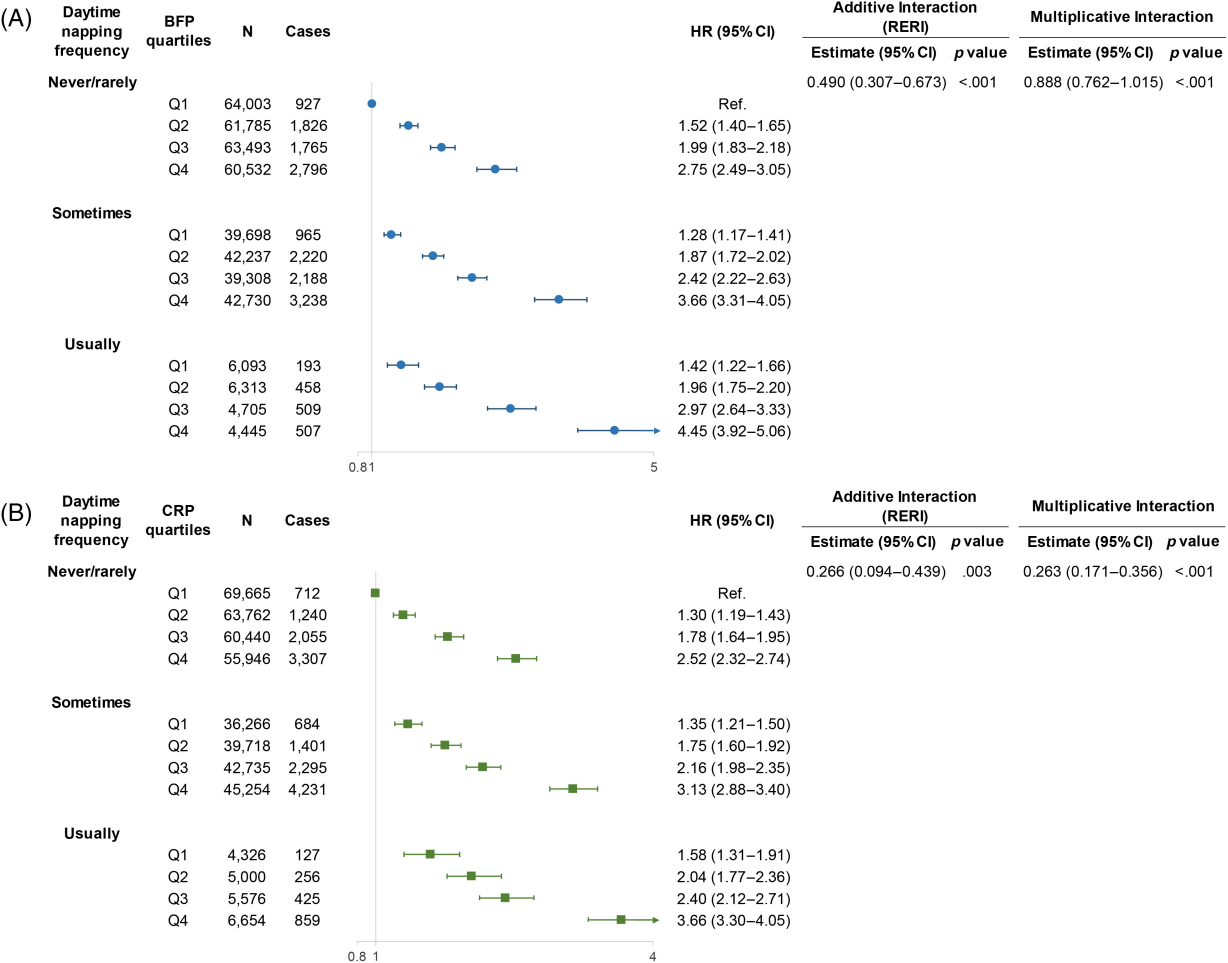
The joint association of daytime napping and quartiles of BFP and CRP in relation to risk of T2D. The joint association of daytime napping and quartiles of BFP (A) and CRP (B) in relation to risk of T2D. Models were adjusted for sex, age (continuous), race, assessment center, Townsend deprivation index (continuous), family income, BMI, smoking status, alcohol consumption status, physical activity (MET‐minutes, continuous), healthy diet, family history of diabetes, antihypertensive medication use, cholesterol medication use, aspirin use, and nonaspirin NSAID use. Abbreviations: BFP, body fat percentage; BMI, body mass index; CRP, C‐reactive protein; MET, metabolic equivalent task; NSAIDs, nonsteroidal anti‐inflammatory drugs; RERI, relative excess risk due to interaction; T2D, type 2 diabetes.

Next, we examined the additive and multiplicative interactions between daytime napping and CRP levels and between daytime napping and BFP on the risk of T2D. Figure 3 shows the associations between daytime napping and risk of T2D, stratified by CRP and BFP quartiles, with those in both nonnappers and the lowest CRP/BFP quartile as the reference group. We found an additive and multiplicative effect for daytime napping and adiposity on the risk of incident T2D (RERI = 0.490, 95% CI: 0.307–0.673; *p* for multiplicative interaction = .009; Figure 3A). Within each daytime napping frequency stratum, there was an increase in the strength of the association with increasing BFP, and the highest risk of incident T2D was observed in habitual nappers in the highest quartile of BFP (HR = 4.45, 95% CI: 3.92–5.06). A similar pattern was observed for CRP, and the highest risk of incident T2D was observed in habitual nappers in the highest quartile of CRP (HR = 3.66, 95% CI: 3.30–4.05). Although the multiplicative interaction between daytime napping and CRP was not statistically significant (*p* for multiplicative interaction = .089), a similar additive interaction (RERI = 0.266, 95% CI: 0.094–0.439) was observed **(**Figure 3B **)**. These results remained materially unchanged after performing several sensitivity analyses (Supplementary Figures S2–S5 in Appendix S1).

## 4 DISCUSSION

In this large‐scale prospective cohort study, we revealed that both a higher frequency of daytime napping and higher levels of CRP and BFP were associated with a higher risk of incident T2D, independent of traditional risk factors for T2D. We also found that the association between daytime napping and incident T2D was substantially augmented by BFP and CRP. When the cohort was stratified by CRP or BFP, the HRs for T2D associated with increasing daytime napping frequencies were almost three times as strong in those with high compared with low levels of CRP or BFP.

Accumulating evidence has indicated U‐shaped relationships between total/nighttime sleep duration and cardiometabolic risks.^29^, ^30^ The associations with daytime napping are less well understood. A dose–response meta‐analysis^31^ of Asian and Western populations showed a J‐curve relationship between nap duration and the risk of diabetes or metabolic syndrome. However, this analysis was mainly based on case–control studies, and thus, temporal relationships could not be determined. The current study prospectively evaluated the effects of napping on T2D risk and demonstrated a positive association between daytime napping frequency and the risk of incident T2D, which is in agreement with the findings of previous studies.^4^, ^5^, ^6^, ^32^ Prospective studies with data on objective measures for napping are needed to examine the association further. Intriguingly, a higher impact of daytime napping on the risk of incident T2D was observed among women and younger individuals (<55 years), which was in line with the results from a cross‐sectional study in a community in China that revealed that in the subgroup analysis stratified by sex and age, the associations between the duration of daytime napping and the prevalence of metabolic syndrome were only significant among females younger than 60 years.^33^ It is likely that women in this age group are experiencing menopause, which causes major hormonal changes and is associated with an adverse metabolic profile that increases the risk of diabetes.^34^ We also observed that the positive association between daytime napping and T2D risk differed when stratified by ethnicity. There was a somewhat stronger association among White participants, in keeping with results from a previous study^35^ that suggested that the detrimental effect of excessive sleep was significant only among non‐Hispanic Whites. These findings highlight the importance of understanding differential pathways linking napping and T2D risk across racial/ethnic groups^36^, ^37^ and, in particular, what cultural or behavioral factors may exacerbate or alleviate the potential effects of daytime napping.

More important, we also revealed that a high napping frequency combined with higher levels of BFP/CRP was associated with an approximately threefold increased risk of T2D compared with nonnappers with low levels of BFP/CRP, indicating that adiposity and inflammation could modify the relationship between daytime napping and T2D risk. Several previous observational studies have demonstrated positive associations between daytime napping and obesity and inflammation, which could enhance the development of diabetes. A cross‐sectional study reported that every 1‐h increase in the duration of afternoon napping increased the odds ratio of obesity 1.23‐fold for males and 1.29‐fold for females.^38^ Another cross‐sectional study also found that daytime napping accounted for a 10.4% increase in obesity prevalence and a 7.1% increase in abdominal obesity prevalence.^39^ In addition, several human studies have reported a positive association between daytime napping and increased inflammatory markers, including CRP and interleukin‐6.^10^, ^11^, ^40^ Nevertheless, there is limited evidence concerning the effects of additive associations between napping and adiposity or inflammation on the risk of incident T2D. Among older adults, a British study^41^ and a Chinese study^17^ reported that several adiposity indices, including BMI, waist circumference, waist‐to‐hip ratio, and waist‐to‐height ratio, significantly mediated the association between napping and T2D risk. However, whether BFP can moderate the relationship between daytime napping and diabetes is less clear. Thus, we filled this gap by prospectively analyzing the association of napping frequency with the risk of T2D and assessing whether BFP and CRP levels might modify the association.

There are several potential explanations for the additive effect of daytime napping with adiposity on the development of T2D. First, daytime napping is considered a compensatory behavioral response to inadequate nocturnal sleep,^42^ which leads to increases in hunger, appetite, and hedonic food intake through endocrine and decision‐related mechanisms.^43^, ^44^ In addition, sleep deprivation could alter neurohormone levels that regulate eating behaviors, leading to an increase in appetite‐stimulating hormones and a decrease in leptin levels.^45^ Second, long naps are directly associated with a prolonged duration of time in bed, which could impair energy homeostasis through various possible mechanisms, including poor sleep quality at night, a less physically active lifestyle, unhealthy dietary habits, and disturbances of circadian rhythm, thereby causing fat deposition and further leading to a higher risk of T2D.^46^ Third, both increased fat mass and awakening from daytime naps, especially prolonged naps, and the combined effects of these two factors could more significantly increase sympathetic activity and lead to disruption of the sympatho‐vagal balance, resulting in activation of the renin‐angiotensin system and subsequent decrease in secretion of pancreatic β cells and glucose dysregulation.^47^, ^48^ Finally, daytime napping has been associated with increased inflammation, which also plays a direct role in obesity‐induced insulin resistance^11^, ^49^; thus, inflammation may act as a link between daytime napping, adiposity and T2D. Regarding inflammation in the link between daytime napping and the risk of incident T2D, it has been suggested that sleep deprivation, which is usually accompanied by napping, is related to the activation of the autonomic nervous system and increased catecholamines and subsequently stimulates the release of inflammatory mediators.^50^

## 5 STRENGTHS AND LIMITATIONS

The current study has several strengths, including the large sample size of UK Biobank participants, the prospective design, the well‐validated measures of BFP and CRP levels, the consistent results in several sensitivity analyses, and the assessment of a wide range of covariates, including lifestyle factors, dietary habits, family history, and medication use, which allowed for rigorous confounding adjustment. More important, our findings added evidence concerning the additive effect of BFP and CRP on the detrimental association between daytime nap frequency and increased risk of T2D.

Nevertheless, several limitations should be considered when interpreting our findings. First, the frequency of daytime napping was self‐reported rather than objectively measured, which might lead to misclassification error. In addition, the assessment of daytime napping was qualitative, and information on napping duration is lacking in the UK Biobank. Further large‐scale studies using more accurate and detailed measurements, such as actigraphy and polysomnography, to assess napping are needed. Second, some incident T2D cases were based on secondary diagnosis; thus, the time of T2D incidence that was used in this study may be later than the actual onset time in this study. However, we obtained similar results when using logistical models (data not shown). Finally, causality cannot be determined because of the observational nature of this study, and randomized clinical trials are needed to verify our findings. However, we deemed that this criticism could be assuaged to some extent, given that several previous prospective cohort studies and Mendelian randomization have consistently reported positive associations of daytime napping with obesity and T2D but not vice versa,^4^, ^5^, ^7^, ^9^, ^33^, ^34^, ^42^, ^51^, ^52^, ^53^ supporting the causal link being inferred.

## 6 CONCLUSION

In summary, the present prospective population‐based cohort study of 435 342 people indicates that a higher frequency of daytime napping is associated with an increased risk of incident T2D, and such relations are modified by BFP and CRP levels. These findings may have implications for the development of T2D prevention strategies targeting the reduction in BFP and CRP levels among older adults with napping habits.

## AUTHOR CONTRIBUTIONS

Rui Zhou designed the study, performed the statistical analysis, interpreted the data, and drafted and critically revised the manuscript; Hao‐Wen Chen helped perform the analysis with constructive discussions and contributed to the interpretation of the results; Yi‐Ning Huang, Qi Zhong, and Fu‐Rong Li contributed to data preparation; Rui‐Dian Huang, Hua‐Min Liu, Jia‐Zhen Zheng, and Jia‐Wen Xu contributed to the interpretation of the results and critical revision of the manuscript; Xian‐Bo Wu conceived and designed the study and contributed to the supervision and project administration of the manuscript. All authors approved the final manuscript. The corresponding author had full access to all of the data in the study and takes responsibility for the integrity of the data and the accuracy of the data analysis.

## CONFLICT OF INTEREST STATEMENT

No potential conflicts of interest relevant to this article were reported.

## Supporting information


**Appendix S1.** Supporting Information

## Data Availability

The data of this study can be requested from the UK Biobank (https://www.ukbiobank.ac.uk/).
