# Supplementary material for: The association between daytime napping and risk of type 2 diabetes is modulated by inflammation and adiposity: Evidence from 435 342 UK‐Biobank participants
Source: J Diabetes. 2023 Apr 13;15(6):496–507. doi: 10.1111/1753-0407.13387 (PMC10270744; doi:10.1111/1753-0407.13387)
Supplement: Supplementary file 1 — Appendix S1. Supporting Information [file JDB-15-496-s001.docx]

**Supplementary materials**

Supplementary Table 1. Definitions of prevalent diabetes and incident T2D.

|  | Source and definition | UK Biobank field code |
| --- | --- | --- |
| Diabetes at baseline [Both “possible” diabetes (T1D, T2D and other types of diabetes) and “probable” diabetes (T1D, T2D and other types of diabetes) were excluded at baseline.] | Self-reported at baseline^1^;  Medication for diabetes at baseline 1;  ICD9: 250, 6480 (if incident time of diabetes ≤ Date of attending assessment center);  ICD10: E10, E11, E12, E13, E14, O24 (if incident time of diabetes ≤ Date of attending assessment center). | 20002, 20003, 2976, 6153, 6177  41271, 41281, 41270, 41280, 53, 191, 40000 |
| Incident T2D | ICD 10: E11 | 41270, 41280 |

1. Eastwood S, Mathur R, Atkinson M, et al. Algorithms for the Capture and Adjudication of Prevalent and Incident Diabetes in UK Biobank. *PLoS One*. 2016;11:e0162388. doi:10.1371/journal.pone.0162388

Supplementary Table 2. The associations between daytime napping and glucose, HbA1c, BFP or CRP levels.

|  | | Model 1 | | |  | Model 2**^*^** | | |
| --- | --- | --- | --- | --- | --- | --- | --- | --- |
|  |  | Beta coefficient | 95% CI | *P* value |  | Beta coefficient | 95% CI | *P* value |
| **Glucose levels** | |  |  |  |  |  |  |  |
|  | Never/rarely | Ref. |  |  |  | Ref. |  |  |
|  | Sometimes | 0.021 | 0.016, 0.026 | <0.001 |  | 0.006 | 0.001, 0.011 | 0.019 |
|  | Often | 0.037 | 0.025, 0.048 | <0.001 |  | 0.165 | 0.005, 0.028 | 0.004 |
| **HbA1c levels** | |  |  |  |  |  |  |  |
|  | Never/rarely | Ref. |  |  |  | Ref. |  |  |
|  | Sometimes | 0.373 | 0.344, 0.401 | <0.001 |  | 0.132 | 0.104, 0.160 | <0.001 |
|  | Often | 0.653 | 0.589, 0.717 | <0.001 |  | 0.230 | 0.168, 0.293 | <0.001 |
| **BFP levels** | |  |  |  |  |  |  |  |
|  | Never/rarely | Ref. |  |  |  | Ref. |  |  |
|  | Sometimes | 1.147 | 1.107, 1.186 | <0.001 |  | 0.297 | 0.272, 0.322 | <0.001 |
|  | Often | 1.600 | 1.512, 1.687 | <0.001 |  | 0.483 | 0.427, 0.538 | <0.001 |
| **CRP levels** | |  |  |  |  |  |  |  |
|  | Never/rarely | Ref. |  |  |  | Ref. |  |  |
|  | Sometimes | 0.403 | 0.376, 0.430 | <0.001 |  | 0.178 | 0.152, 0.204 | <0.001 |
|  | Often | 0.760 | 0.700, 0.819 | <0.001 |  | 0.403 | 0.345, 0.461 | <0.001 |

Model 1 was adjusted for sex and age (continuous).

Model 2 was additionally adjusted for race, assessment center, Townsend deprivation index (continuous), family income, BMI, smoking status, alcohol consumption status, physical activity (MET-minutes, continuous), healthy diet, family history of diabetes, antihypertensive medication use, cholesterol medication use, aspirin use, and nonaspirin NSAID use.

^*^ In glucose analysis, model 2 was additionally adjusted for fasting time.

Supplementary Table 3. The hazard ratios of daytime napping for T2DM after excluding participants with glucose levels ≥ 7.0 (mmol/L) at baseline.

|  | N | Events | Model 1 | Model 2 |
| --- | --- | --- | --- | --- |
| Never/rarely | 246,664 | 6,575 | Ref. | Ref. |
| Sometimes | 161,333 | 7,734 | 1.62 (1.57-1.68) | 1.28 (1.24-1.32) |
| Usually | 21,103 | 1,475 | 2.12 (2.00-2.25) | 1.46 (1.38-1.55) |
| *P* value |  |  | <0.001 | |

Model 1 was adjusted for sex and age (continuous).

Model 2 was additionally adjusted for race, assessment center, Townsend deprivation index (continuous), family income, BMI, smoking status, alcohol consumption status, physical activity (MET-minutes, continuous), healthy diet, family history of diabetes, antihypertensive medication use, cholesterol medication use, aspirin use, and nonaspirin NSAID use.

**P* values were from Chi-square tests examining the difference in hazard ratio across groups.

Supplementary Table 4. The hazard ratios of daytime napping for T2DM after excluding participants with HbA1c levels ≥ 48 (mmol/mol) at baseline.

|  | N | Events | Model 1 | Model 2 |
| --- | --- | --- | --- | --- |
| Never/rarely | 248,625 | 6,520 | Ref. | Ref. |
| Sometimes | 162,509 | 7,559 | 1.60 (1.55-1.66) | 1.27 (1.23-1.31) |
| Usually | 21,275 | 1,457 | 2.12 (2.00-2.25) | 1.47 (1.39-1.56) |
| *P* value |  |  | <0.001 | |

Model 1 was adjusted for sex and age (continuous).

Model 2 was additionally adjusted for race, assessment center, Townsend deprivation index (continuous), family income, BMI, smoking status, alcohol consumption status, physical activity (MET-minutes, continuous), healthy diet, family history of diabetes, antihypertensive medication use, cholesterol medication use, aspirin use, and nonaspirin NSAID use.

**P* values were from Chi-square tests examining the difference in hazard ratio across groups.

Supplementary Table 5. The hazard ratios of daytime napping for T2DM after excluding participants who developed T2D within 2 years of follow-up.

|  | N | Events | Model 1 | Model 2 |
| --- | --- | --- | --- | --- |
| Never/rarely | 239,456 | 6,821 | Ref. | Ref. |
| Sometimes | 157,072 | 8,009 | 1.62 (1.57-1.67) | 1.28 (1.24-1.32) |
| Usually | 20,560 | 1,521 | 2.11 (1.99-2.23) | 1.47 (1.39-1.56) |
| *P* value |  |  | <0.001 | |

Model 1 was adjusted for sex and age (continuous).

Model 2 was additionally adjusted for race, assessment center, Townsend deprivation index (continuous), family income, BMI, smoking status, alcohol consumption status, physical activity (MET-minutes, continuous), healthy diet, family history of diabetes, antihypertensive medication use, cholesterol medication use, aspirin use, and nonaspirin NSAID use.

**P* values were from Chi-square tests examining the difference in hazard ratio across groups.

Supplementary Table 6. The hazard ratios of daytime napping for T2DM after excluding participants who regularly took anti-inflammatory drugs.

|  | N | Events | Model 1 | Model 2 |
| --- | --- | --- | --- | --- |
| Never/rarely | 188,164 | 4,962 | Ref. | Ref. |
| Sometimes | 116,571 | 5,438 | 1.62 (1.56-1.68) | 1.28 (1.23-1.33) |
| Usually | 14,512 | 967 | 2.12 (1.97-2.27) | 1.49 (1.39-1.60) |
| *P* value |  |  | <0.001 | |

Model 1 was adjusted for sex and age (continuous).

Model 2 was additionally adjusted for race, assessment center, Townsend deprivation index (continuous), family income, BMI, smoking status, alcohol consumption status, physical activity (MET-minutes, continuous), healthy diet, family history of diabetes, antihypertensive medication use and cholesterol medication use.

**P* values were from Chi-square tests examining the difference in hazard ratio across groups.

Supplementary Table 7. Hazard ratios of daytime napping for T2DM after further adjustment for glucose levels, HbA1c levels, CRP levels, and sleep duration.

|  | Never/rarely | Sometimes | Usually | *P* value |
| --- | --- | --- | --- | --- |
| Events/N | 6,703/228,662 | 7,935/150,659 | 1,546/19,864 |  |
| Model 1+glucose+fasting time | Ref. | 1.26 (1.22-1.30) | 1.51 (1.43-1.60) | <0.001 |
| Events/N | 6,943/237,574 | 8,173/155,795 | 1,578/20,454 |  |
| Model 1+HbA1c | Ref. | 1.25 (1.21-1.29) | 1.49 (1.40-1.57) | <0.001 |
| Events/N | 7,314/249,813 | 8,611/163,973 | 1,667/21,556 |  |
| Model 1+CRP | Ref. | 1.27 (1.23-1.31) | 1.47 (1.39-1.55) | <0.001 |
| Events/N | 7,262/248,840 | 8,490/162,711 | 1,639/21,323 |  |
| Model 1+Sleep duration | Ref. | 1.29 (1.25-1.33) | 1.52 (1.44-1.61) | <0.001 |

Model 1 was adjusted for sex, age (continuous), race, assessment center, Townsend deprivation index (continuous), family income, BMI, smoking status, alcohol consumption status, physical activity (MET-minutes, continuous), healthy diet, family history of diabetes, antihypertensive medication use, cholesterol medication use, aspirin use, and nonaspirin NSAID use.

**P* values were from chi-square tests examining the difference in hazard ratios across groups.

Supplementary Figure 1. Kaplan–Meier survival curves of incident T2D onset over the follow-up stratified by napping frequencies.


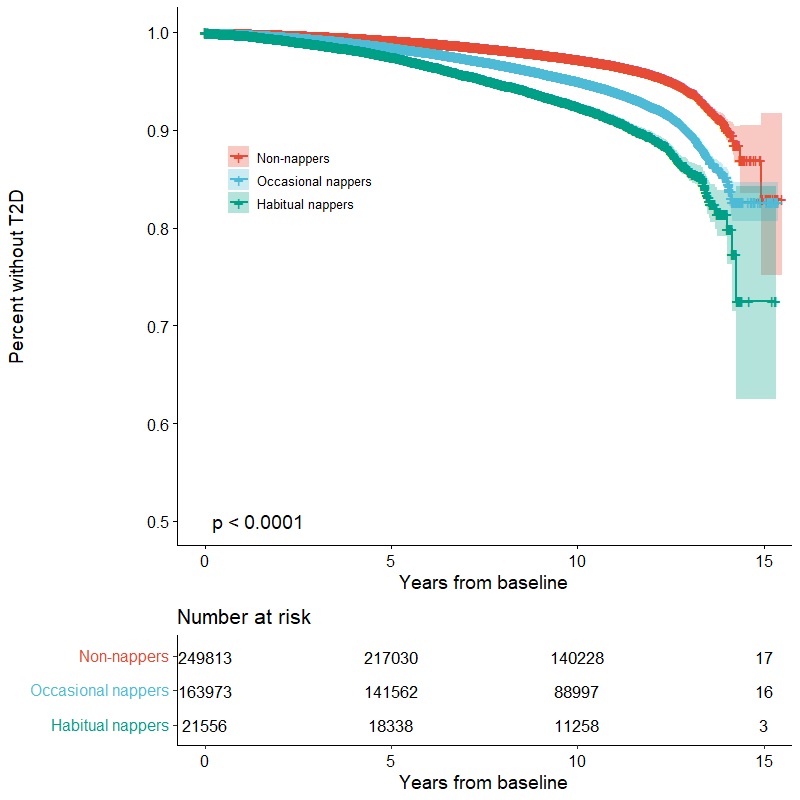


Supplementary Figure 2. The joint association of daytime napping and quartiles of CRP (A) and BFP (B) in relation to risk of T2D after excluding participants with glucose level ≥ 7.0 (mmol/L) at baseline.


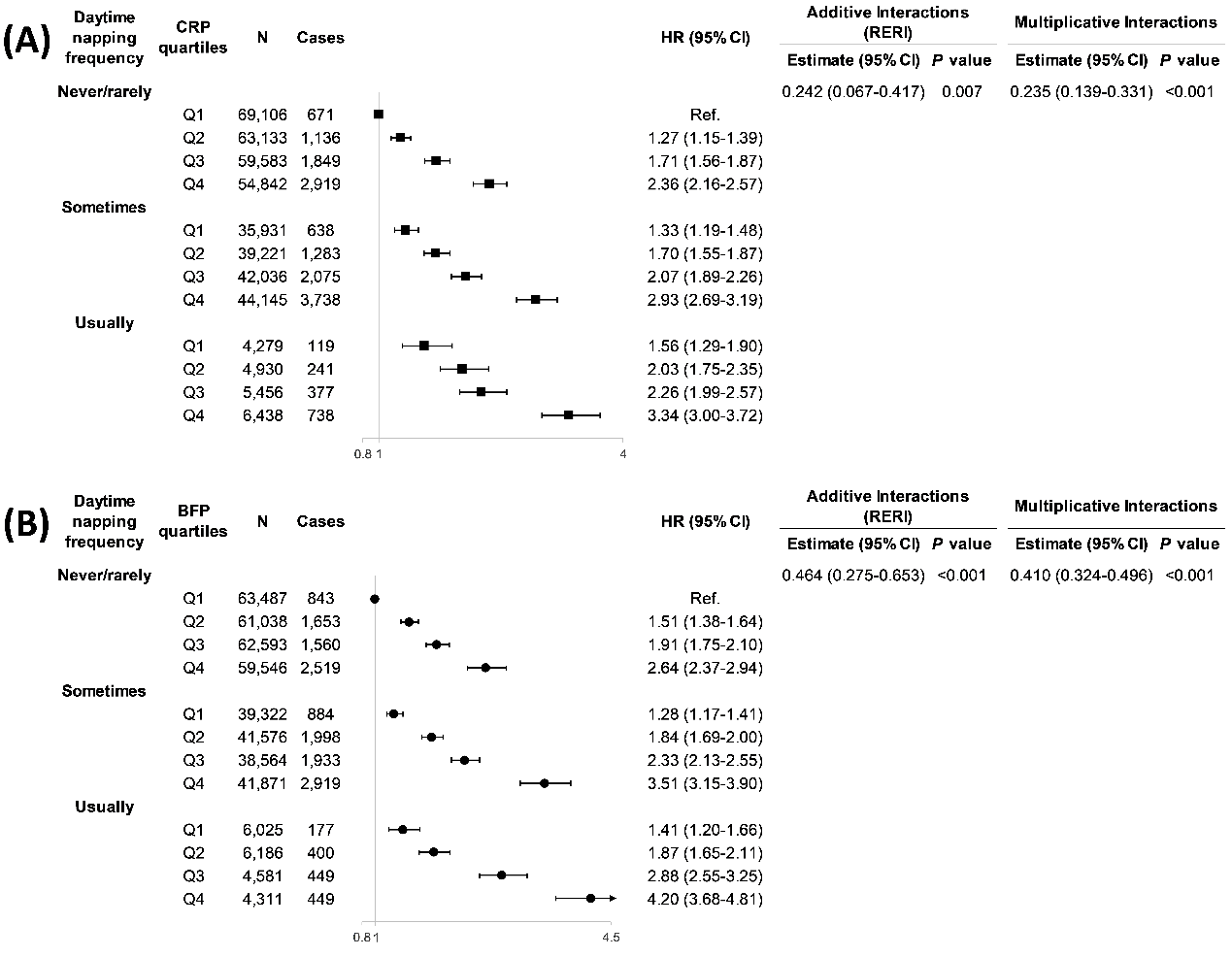


Models were adjusted for sex, age (continuous), race, assessment center, Townsend deprivation index (continuous), family income, BMI, smoking status, alcohol consumption status, physical activity (MET-minutes, continuous), healthy diet, family history of diabetes, antihypertensive medication use, cholesterol medication use, aspirin use, and nonaspirin NSAID use.

Abbreviations: BFP, body fat percentage; CRP, C-reactive protein; T2D, type 2 diabetes; RERI, relative excess risk due to interaction; BMI, body mass index; NSAID, non-steroidal anti-inflammatory drugs.

Supplementary Figure 3. The joint association of daytime napping and quartiles of CRP (A) and BFP (B) in relation to risk of T2D after excluding participants with HbA1c levels ≥ 48 (mmol/mol) at baseline.


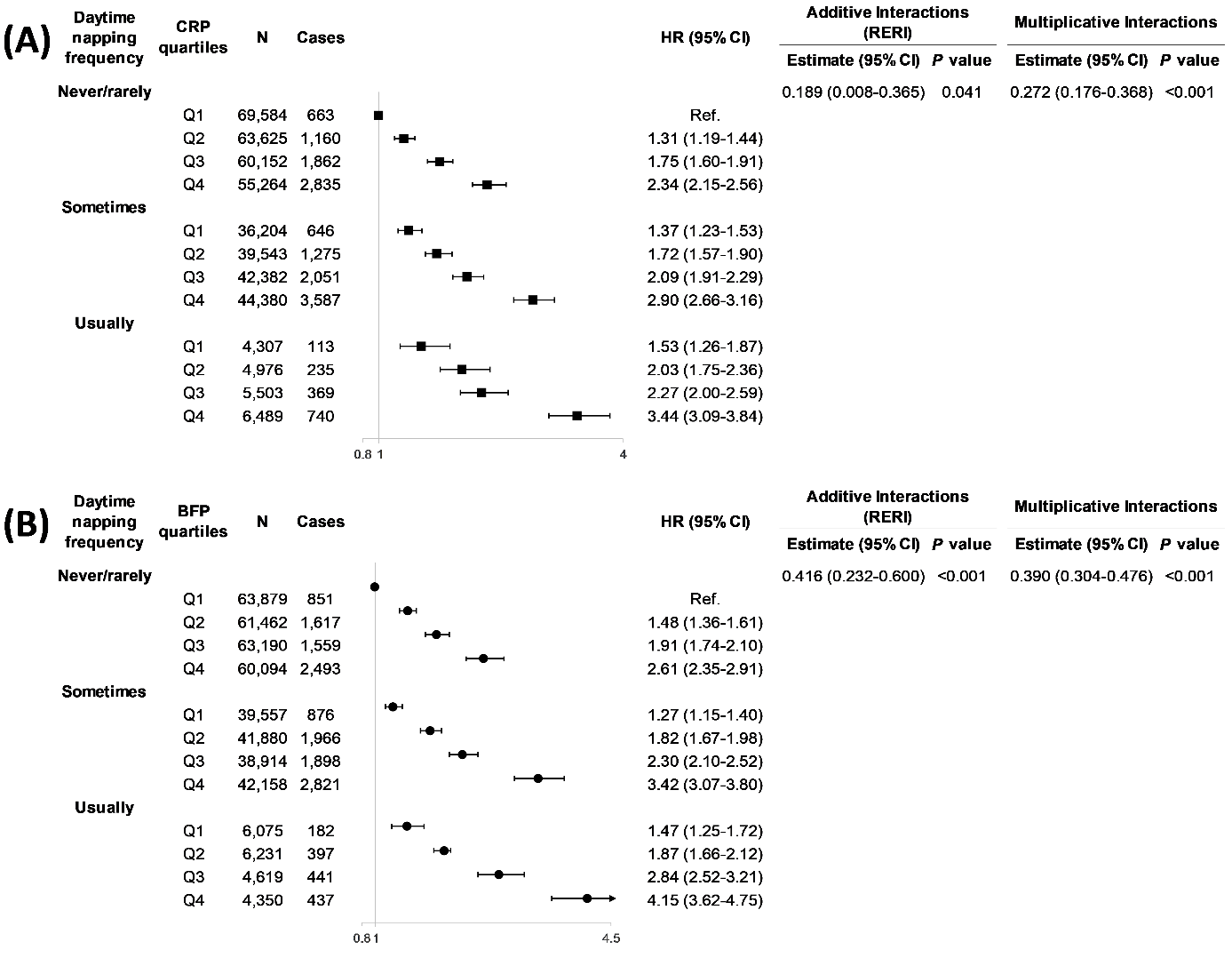


Models were adjusted for sex, age (continuous), race, assessment center, Townsend deprivation index (continuous), family income, BMI, smoking status, alcohol consumption status, physical activity (MET-minutes, continuous), healthy diet, family history of diabetes, antihypertensive medication use, cholesterol medication use, aspirin use, and nonaspirin NSAID use.

Abbreviations: BFP, body fat percentage; CRP, C-reactive protein; T2D, type 2 diabetes; RERI, relative excess risk due to interaction; BMI, body mass index; NSAID, non-steroidal anti-inflammatory drugs.

Supplementary Figure 4. The joint association of daytime napping and quartiles of CRP (A) and BFP (B) in relation to risk of T2D after excluding participants participants who developed T2D within 2 years of follow-up.


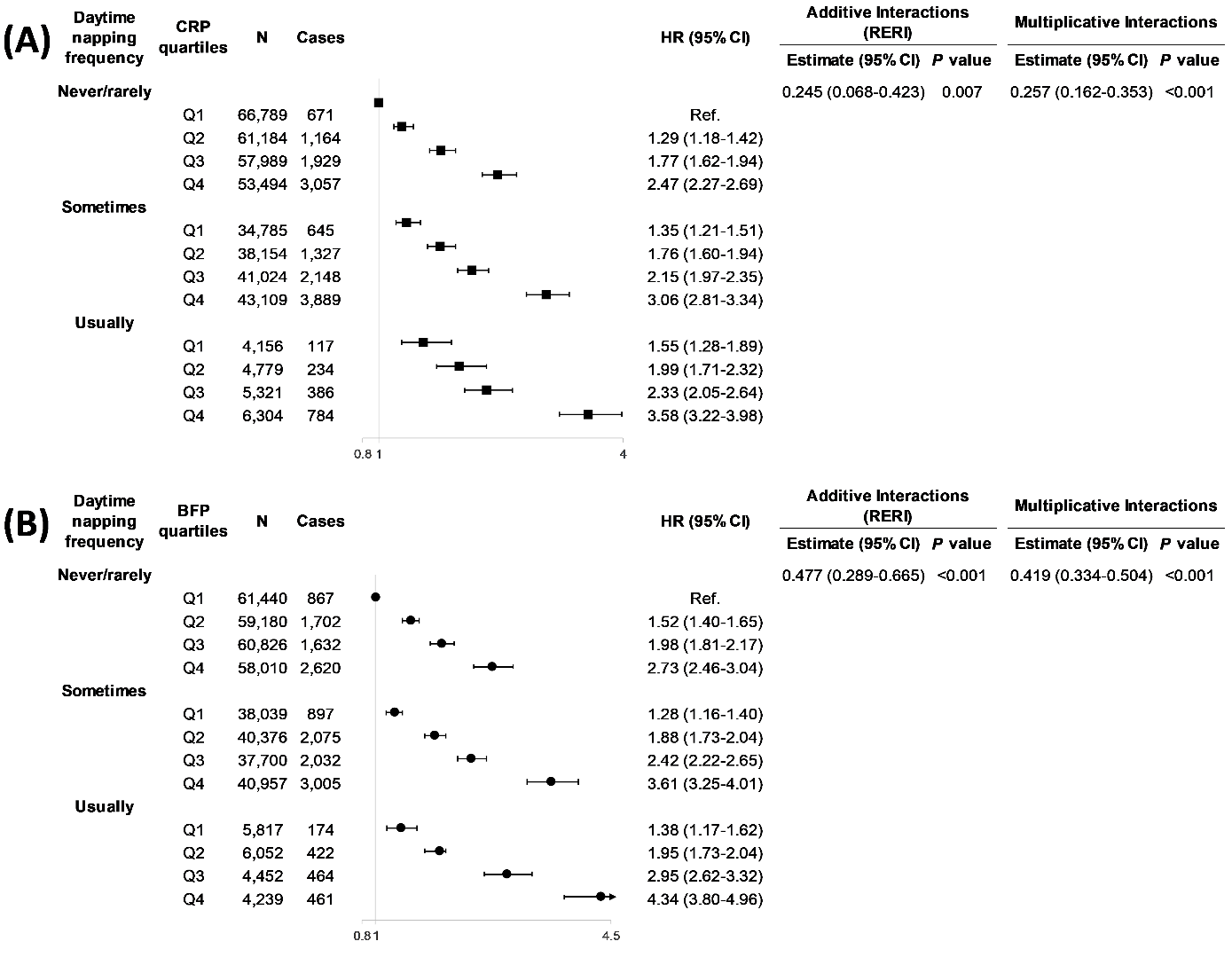


Models were adjusted for sex, age (continuous), race, assessment center, Townsend deprivation index (continuous), family income, BMI, smoking status, alcohol consumption status, physical activity (MET-minutes, continuous), healthy diet, family history of diabetes, antihypertensive medication use, cholesterol medication use, aspirin use, and nonaspirin NSAID use.

Abbreviations: BFP, body fat percentage; CRP, C-reactive protein; T2D, type 2 diabetes; RERI, relative excess risk due to interaction; BMI, body mass index; NSAID, non-steroidal anti-inflammatory drugs.

Supplementary Figure 5. The joint association of daytime napping and quartiles of CRP (A) and BFP (B) in relation to the risk of T2D after excluding participants who regularly took anti-inflammatory drugs.


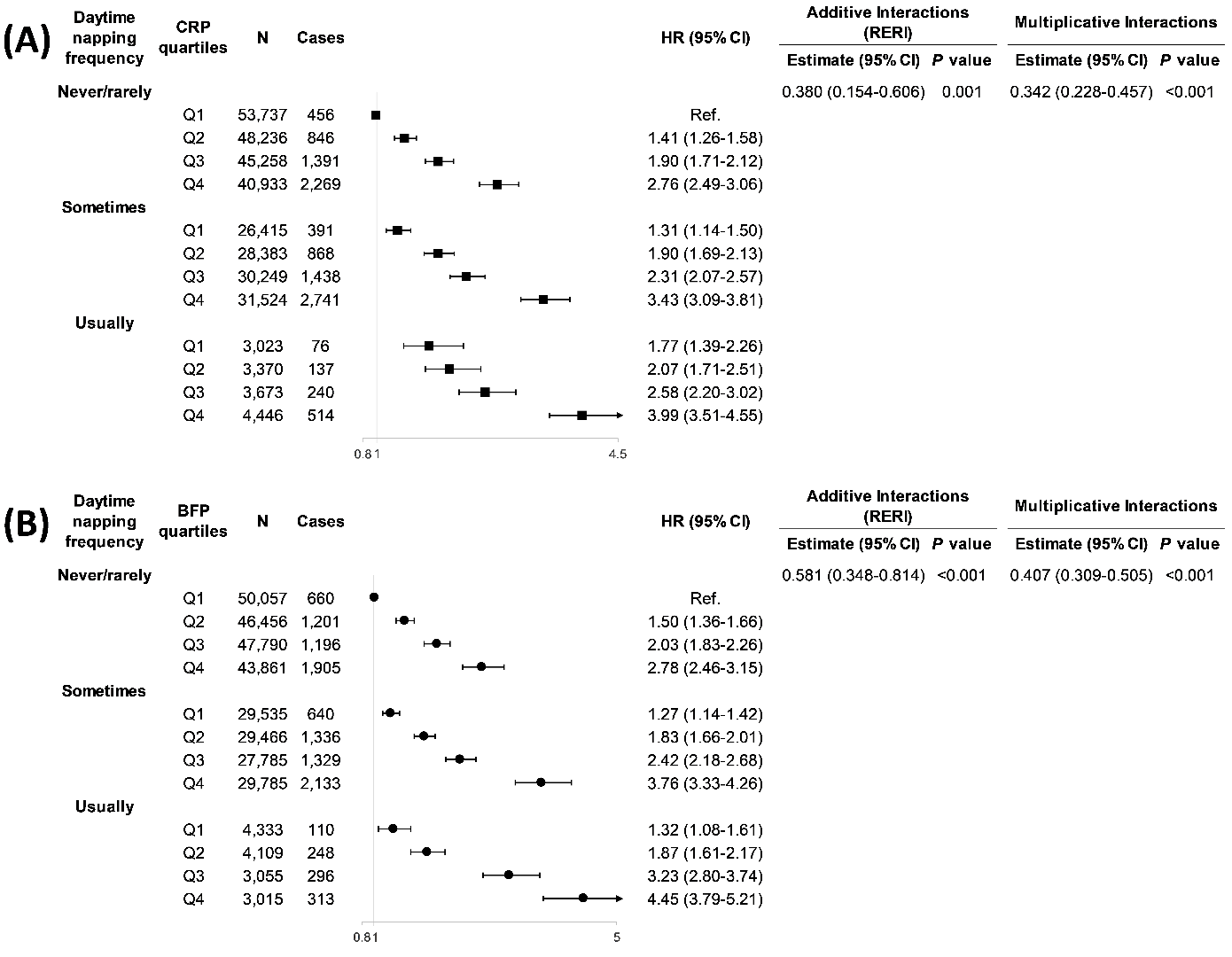


Models were adjusted for sex, age (continuous), race, assessment center, Townsend deprivation index (continuous), family income, BMI, smoking status, alcohol consumption status, physical activity (MET-minutes, continuous), healthy diet, family history of diabetes, antihypertensive medication use, and cholesterol medication use.

Abbreviations: BFP, body fat percentage; CRP, C-reactive protein; T2D, type 2 diabetes; RERI, relative excess risk due to interaction; BMI, body mass index; NSAID, non-steroidal anti-inflammatory drugs.
